# Using telehealth to support end of life care in the community: a feasibility study

**DOI:** 10.1186/s12904-016-0167-7

**Published:** 2016-11-17

**Authors:** Jennifer J. Tieman, Kate Swetenham, Deidre D. Morgan, Timothy H. To, David C. Currow

**Affiliations:** 1 Palliative and Supportive Services, Flinders University, Bedford Park, South Australia Australia; 2 Southern Adelaide Palliative Services, Repatriation General Hospital, Daw Park, South Australia Australia

**Keywords:** Telemedicine, Palliative care, Home care services

## Abstract

**Background:**

Telehealth is being used increasingly in providing care to patients in the community setting. Telehealth enhanced service delivery could offer new ways of managing load and care prioritisation for palliative care patients living in the community. The study assesses the feasibility of a telehealth-based model of service provision for community based palliative care patients, carers and clinicians.

**Methods:**

This study was a prospective cohort study of a telehealth-based intervention for community based patients of a specialist palliative care service living in Southern Adelaide, South Australia. Participants were 43 community living patients enrolled in the Southern Adelaide Palliative Service. To be eligible patients needed to be over 18 years and have an Australian modified Karnofksy Performance Score > 40. Exclusion criteria included a demonstrated inability to manage the hardware or technology (unless living with a carer who could manage the technology) or non-English speaking without a suitable carer/proxy. Participants received video-based conferences between service staff and the patient/carer; virtual case conferences with the patient/carer, service staff and patient’s general practitioner (GP); self-report assessment tools for patient and carer; and remote activity monitoring (ACTRN12613000733774).

**Results:**

The average age of patients was 71.6 years (range: 49 to 91 years). All 43 patients managed to enter data using the telehealth system. Self-reported data entered by patients and carers did identify changes in performance status leading to changes in care. Over 4000 alerts were generated. Staff reported that videocalls were similar (22.3%) or better/much better (65.2%) than phone calls and similar (63.1%) or better/much better (27.1%) than face-to-face. Issues with the volume of alerts generated, technical support required and the impact of service change were identified.

**Conclusions:**

The trial showed that patients and carers could manage the technology and provide data that would otherwise not have been available to the palliative care service.

**Trial registration:**

Australian New Zealand Clinical Trials Registry ACTRN12613000733774 registered on 02/07/2013.

## Background

Despite increasing investment in inpatient palliative care facilities, the vast majority of palliative care will continue to be provided in the home where patients and their families may be cared for by their primary care providers, in shared care arrangements with specialist palliative care services or as patients of the specialist palliative care service [1–3]. Home-based care often covers a period of time from weeks to months, or even years. Most palliative care patients indicate that they would prefer to be cared for, and to die, at home. However, care in the community commonly requires support from family or friends who fulfil a vital caregiver role which enables the patient to remain at home [4, 5]. It also requires flexible and responsive health care provision that provides continuity of care and addresses changing health needs [6]. This can be difficult to deliver within resource-constrained services where contact may be limited to intermittent telephone calls and occasional home visits. As demand for palliative care increases as a result of an ageing population and progressive chronic illnesses, mechanisms that support home-based care need to be explored. Telehealth represents one avenue for investigation, offering more regular engagement through videoconferencing, potentially continuous remote monitoring to highlight changing performance status, and immediate access to resources and information for patients and families. For clinicians and services, a telehealth enhanced service for the community may enable them to allocate their staff resources more appropriately to patients where self-reported symptom needs are high or where unanticipated changes are being identified.

Telehealth uses information and communication technologies to capture and transmit health data and to deliver services and information [7]. Not all healthcare can be delivered through telehealth, however, many specialties are using or have tested telehealth to augment or replace some aspects of care and it is being delivered in various clinical service settings. Reviews of the feasibility and effectiveness of telehealth suggest that telehealth may offer benefits to patients with a range of conditions but that various factors may influence its usefulness and effectiveness such as the severity of the condition or the disease trajectory and how the intervention works within the service delivery model [8–11]. Reviews also indicate that there is satisfaction with the use of telehealth, mainly videoconferencing applications, by patients and by carers [12–14].

Even given the range of potential applications that could be useful within palliative care, there is limited research into the potential role, feasibility and effectiveness of telehealth applications for palliative care delivered into the community. Authors have acknowledged the potential contribution that telehealth could play in palliative care for patients in rural and remote areas [15], the role that mobile technologies could play [16], and opportunities for specific population groups such as paediatrics [17] or lung cancer [18]. However, reviews and trials have also highlighted the need to build the evidence around palliative care telehealth in the community [12–14, 19, 20]. Recent evidence also indicates that provider acceptance of telehealth within palliative care organisations plays a key role in ensuring its uptake and utilisation [21]. As such, there is a need to build the evidence base around the benefits and burdens of telehealth, together with its acceptability to patients and their families and to health providers. Understanding the extent, likelihood, and manner in which such interventions can be implemented as planned and proposed is important in determining the feasibility of telehealth as part of care delivery. This study investigates the feasibility of a telehealth intervention for community based palliative care patients, carers and clinicians (ACTRN12613000733774).

## Methods

The study was conducted in the Southern Adelaide Palliative Care Service (SAPS), South Australia. Ethics approval for the study was granted by the Southern Adelaide Clinical Human Research Ethics Committee on 2 August 2013 (168.13). Five subsequent amendments were sought and all approved.

A Palliative Care Telehealth Research Team (PCTRT) was established to guide the development and implementation of the telehealth model for use by the community team of a specialist palliative care service. Membership of the PCTRT included the Director of the Clinical Service, clinical staff (medicine, nursing, allied health), and researchers with expertise in clinical trial design, health informatics, health services research, and evaluation. A Project Manager was appointed to support the project development. Input was sought and received during concept and module development from service providers, stakeholders, and patients and carers involved with the service. The PCTRT met regularly with the telehealth technical team as well as with the external IT consultant and web provider across the course of the project. As telehealth resources must satisfy the utility and usability criteria of clinicians and consumers not just those of funders and system providers, meetings were held with the clinicians providing direct care to enable input and feedback on the proposals and ongoing management of the telehealth applications. Details on the development of the resources have been previously reported [22].

**Fig. 1:**
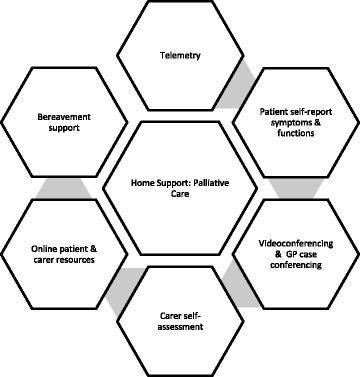
Components of Telehealth model

The components of the palliative care telehealth model to support patient and carer in the home environment and to enhance clinical feedback are outlined in Fig. 1. They included ongoing video-based conferences between service staff and the patient or carer, virtual case conferences with the patient and carer, service staff and the patient’s general practitioner (GP), self-report assessment tools for the patient and carer, and remote activity monitoring.

The study design was a prospective cohort study of a telehealth-based intervention for SAPS palliative care patients based in the community. Patients were invited to participate in the study if they were not in bed more than 50% of the time as indicated by a performance score greater than 40 using Australia-modified Karnofsky Performance Status (AKPS) assessment at the point of entry to the study [23]. Participants needed to be 18 years of age or older. Potential participants who demonstrated inability to manage the hardware or technology (unless living with a carer who could manage the technology) or who were non English speaking without a suitable carer/proxy were excluded. Participants were able to withdraw at any time.

Potentially eligible participants were identified from the SAPS Client List during a weekly screening process. They were approached by the SAPS clinical team, (usually the Clinical Nurse Consultant or Caregiver Network Facilitator) to consider participation in the study. Potential participants were then contacted by a member of the investigating team to discuss participation in the study. An appointment was made to outline the study and obtain consent. Given a lack of familiarity with technology in many cases and potential burden given the stage of illness, a second appointment was routinely made to demonstrate the technology uses and to assist in entering the first set of data with the patient and/or carer.

Participants received a combined telecare and telemonitoring package using an iPad tablet for data entry [24]. The components of the package included:

- structured online video contacts between the patient and the Nurse Clinical Practice Consultant (CPC) and between the carer and the Nurse Clinical Practice Consultant and/or Caregiver Network Facilitator (CNF)
- patient self-assessment and online data entry using the following tools—AKPS [23], Assessment of Quality of Life (AQoL) [25] and Symptom Assessment Scale (SAS) [26]. Alerts were sent to the relevant health professional if scores breached pre-specified thresholds
- carer self-report using the service’s carer needs self-assessment tool, Caregiver Assessment Questionnaire-Caregiver Network Service (CAQ-CNS) which was developed for use in the community facilitator/caregiver pilot study and then adopted for use in SAPS [27], and assessment of the patient’s function by the caregiver using the AKPS [23]. Alerts were sent to the relevant health professional if scores breached pre-specified thresholds
- health care utilisation monitoring via a self-report electronic diary
- planned responses if self-reported assessments exceeded pre-defined thresholds generally identified by alerts. Responses included face to face videoconference, home visit and/or virtual case conference with the family, GP and palliative care service
- access to appraised and structured online information resources for patients and carers were included in project specific pages held on the CareSearch website [28], and activity and weight monitoring using FitBit®™ technology [29].

Applications enabled self-reported data to be entered and stored in the CareSearch website [28]. SAPS clinicians providing direct care to study participants received an iPad for project use and training in the functionality of the iPad and the individual applications. Following each video interaction with a client, the participating clinicians completed a brief assessment on the quality of the technology and the significance of the interaction. Clinician perceptions on the telehealth project were collected in focus groups and interviews and have been reported elsewhere [30].

## Results

Forty-three community participants received active services between 6 June 2013 and 31 July 2014. Forty-one patients on the trial had a cancer diagnosis. On average, patients spent 128.9 days (range: 17 to 415) on the program. The average age of patients was 71.6 years (range: 49 to 91 years). Thirty-one patients were aged 65 years or older with 12 of these being over 80 years. Of the 43 participants, 38 were patient-carer dyads and five participants had no carer. Seven participants lived alone with two having visiting rather than resident carers. Two of the carers were siblings, three were daughters and the remaining 33 were partners or spouses. Seventeen of the patients were women (39.5%) and 26 (60.5%) were men. Only the carer was consented in one dyad as the patient’s level of cognition was insufficient for self-reporting of symptoms and function.

Data entry shows that patients and carers were able to use the technology and did self-report using the applications. Table 1 reports the number of times the various tools were accessed for data entry. As the SAS comprises seven separate symptom reports, the number of alerts can exceed the number of times the scale was accessed as a patient may be reporting high scores for multiple symptoms. This is similar for the CAQ-CNS which also comprises multiple items that could lead to an alert.

**Table 1:** Number of times tools accessed by patients and carers and number of alerts arising

Application (Tool)	Scale access	Alerts arising
Australian-modified Karnofsky Performance Scale (Function)	1058	611
Symptom Assessment Scale (Symptoms)	3009	4386
Caregiver Assessment Questionnaire-Caregiver Network Service (Carer self-assessment)	263	283

Alerts were generated by the system when data entered by the patient or by the carer breached pre-determined thresholds. For example, the threshold for the SAS was ≥ 5 and ≤ 70 for the AKPS. There were 611 alerts arising from changes in the AKPS across the study and 4386 alerts generated through SAS. Of the 4386 alerts arising from the SAS, pain and fatigue were the most commonly reported symptoms generating 726 and 1205 alerts respectively. Data showed that patients were also using the ‘Other’ category in SAS to identify symptoms not included in the scale that were causing them concern. Thirty-five of 38 carers completed the CAQ-CNS at least once (92.1%). A score above seven on any item in the CAQ-CNS was classified as an alert.

Each of the tools had a specified frequency for patient and/or carer self-report. Patients and carers were instructed to complete the AKPS on a weekly basis. On average, patients entered data 1.25 times per week, or 25% more frequently than expected. The SAS was expected to be competed daily however, patients entered data less frequently, on average 0.73 times daily or 27% less than expected. Patients generally entered data more closely to what was required at the beginning of their time on the program (i.e., the first 2 weeks). Of those who died while receiving the telehealth intervention (*n* = 15), the deceased had on average stopped entering data 16 days before death. On average, carers entered data 0.60 times per week. The data shows that the actual patterns of self-reporting by patients and carers varied from the expected rates of data entry.

In total, there were 180 recorded contacts made with patients, carers or both arising from scheduled calls or from contacts made in response to alerts. The primary mode of contact was video-conferencing (56.7%), followed by phone call (29.4%), then face-to-face (13.9%). Contact was most often made with both carers and patients (44.9%), followed by carer only 35.9%) and patient only (19.1%). Scheduled contact (67.3%) was more common than unscheduled contact (32.7%). Fifty seven unscheduled contacts were made. Reasons for unscheduled contacts were: symptom trigger alerted through SAS entry (25.5%), followed by message from patient (20.0%), decline in functional status alerted through AKPS entry (21.8%), caregiver trigger alerted through CAQ-CNS entry (18.2%) or non-completion of any data for 2 days (14.6%). This indicates that clinical responses are being made to data being entered by the patient.

With respect to the outcomes of clinical contacts, 111 notes were recorded in clinical records. A change in medication was the most common noted outcome. Data indicate that four admission recommendations were made following a video call (Table 2).

**Table 2:** Reported outcome of service contact by type of contact

	Phonecall	Videocall	Face to face
Medication change	10	15	11
Admission recommendations	1	4	–
Other	4	9	6
No change	12	33	6
Total	27	61	23

The clinical staff made 121 ratings on conducting clinical assessments using videocalls compared to a phone call and 111 ratings comparing videocalls to face to face. The nurses reported that videocalls were similar to (22.3%) or better/much better than (65.2%) phone calls. Videocalls were also reported to be similar to (63.1%) or better/much better (27.1%) than face-to-face contacts (Table 3).

**Table 3:** Comparison of videocall to normal practice

Video review	Much worse	Worse	Similar	Better	Much better
Compared to phone call (*N* = 121)	4 (3.3%)	11 (9.1%)	27 (22.3%)	59 (48.7%)	20 (16.5%)
Compared to face to face (*N* = 111)	3 (2.7%)	7 (6.3%)	70 (63.1%)	30 (27.0%)	1 (0.1%)

*Abbreviations*: *N* number

Nurses entered responses about clinical outcomes in their clinical notes and identified instances in which telehealth made a difference to clinical practice. Nurses also indicated that technology had been very effective in enabling the patient or carer to be reassured (57.3%), quicker problem management (23.2%), identification of problems that may not have been recognised (20.5%), ability to resolve issues that would have previously required a home visit (16.8%), and ability to share information with other health professionals (7.6%).

Patient and carer attitudes and responses to the telehealth initiative were sought through interviews and are being reported separately.

## Discussion

### Feasibility of telehealth

This study sought to determine if a telehealth enhanced community service for palliative care patients was feasible. The trial showed that patients and carers, including patients over 80 years, could manage the technology and provide data that would otherwise not have been available to the palliative care services. Self-reported data entered by patients and carers did identify changes in performance state and in symptom distress triggering alerts to the service provider. Scheduled videocall contacts and contacts made in response to triggers led to changes in care. Clinicians reported that the quality of the telehealth contact was acceptable and in most case comparative to current modes of contact. These findings suggest that telehealth approaches to support community-based palliative care patients are feasible and valuable for clinical care.

For palliative care patients, where changes in symptoms are not uncommon and relatively high scores can also be expected at some points or with some co-morbidities, telehealth provides a window into the community patient’s status. There are some suggestions that those receiving care based in the community may not always have the same outcomes as inpatient care. For example, summary data from the Australian Palliative Care Outcomes Collaboration for the period 2010 to 2015 notes that inpatients are more likely to have a shorter time in the unstable phase than those in the community (86 to 77%). It also reports that those being cared for in inpatient settings are more likely to have no pain or mild pain at the end of a period of care provision than those receiving community based care (89 to 82%) [31]. Telehealth may provide a mechanism to reduce this variability between inpatient and community settings by enabling more consistent monitoring and more reactive management of symptoms.

Telehealth may also enable a more equitable management of palliative care resources by enabling clinical time to be directed to those in the community with the greater need. The current model results in limited contact between visits; the telehealth intervention captures escalating clinical need that otherwise remains hidden until a crisis arises. Given policy directions and consumer desires to remain at home, telehealth may also assist in managing the projected increased demand for palliative care services associated with ageing, progression of chronic diseases and more timely referral to palliative care in the disease trajectory. Proactively monitoring symptoms, rather than responding to crises, could enable teams to deliver care in a more efficient manner by targeting community visits where symptom change or carer burden are being recorded.

### Alerts and data reporting

While this study has shown that telehealth enables self-reporting of symptoms and the generation of alerts, it also highlights complexity around the role of alerts. Alerts did not necessarily result in a clinical contact or response. In many instances they appeared to be providing clinical information rather than acting as a clinical trigger. Moreover, even though the alerts were being triggered in accordance with agreed clinical thresholds, the number of ongoing alerts proved burdensome. For example, breathlessness scores may increase and may remain high as a patient’s disease progresses. Such alerts will continue, even if the symptom cannot be fully corrected. Clinicians noted that their clinical knowledge of the patient also informed their interpretation of routinely high symptom alerts. Their feedback suggests that review of the agreed clinical thresholds and a more sophisticated dashboard that would place an alert in the context of the patient’s status over time could assist in prioritising and responding to alerts. Addressing the issue of burdensome alerts is important as in other systems, particularly computerized provider order entry (CPOE) and clinical decision support (CDS), alert fatigue and ignoring alerts tends to increase with growing exposure to alerts and heavier use of the systems [32, 33].

Variability in the patterns of self-reported data entry suggests that the impact of compliance needs to be considered in assessing feasibility. Patient compliance issues in home-based telehealth studies have been recognised and reported and it is not uncommon to find that compliance with data entry will diminish over time [34]. User training and user support have been identified as mechanisms to mitigate against this effect [34]. However, it is worth considering that in a palliative care context where changes and decline should be anticipated that variability in participation and compliance with the data entry regime should also be anticipated [35]. Further work may help to identify the optimal point at which the introduction of telehealth in community-based care should occur and whether changes in data entry may also be a further indicator of a changing patient condition. The possibility of normalising telehealth applications by introducing them in ambulatory clinic as a means of familiarising patients and caregivers with their functions and applications may also be worthwhile.

### Telehealth support requirements

Implementing telehealth depends on a sophisticated infrastructure given current interoperability considerations, and privacy and security concerns within the health system. These add to the complexity of planning for service delivery [36, 37]. Providing a telehealth enhanced palliative care service required dedicated technical resource to be made available to support clinical service delivery. However, some of these technical support needs were related to the capture of research and evaluation data to assess feasibility of the approach rather than the service delivery itself. Others related to meeting the specific processes and regulations needed to allow some level of integration with clinical record systems in the health service. While privacy and security are important considerations for technology enabled solutions they may also limit flexibility in delivery of community services if the patient’s personal IT resources are not able to be networked to the health service.

Technology upgrades and cyber threats outside the control of the project also influenced the project. For example, a transient security threat for technology platforms, The Heartbleed bug had a significant, albeit brief, impact on this project. Heartbleed is a weakness in the encryption security of programs such as email, internet sites and for specific programs such as FitBit®™. It has the potential to allow hackers to access data from a range of sites and services. As a security measure, FitBit®™ forced a reset on all passwords on their products. This required a manual reset of all FitBit®™ passwords on the tablets. Each participant was contacted and a time made for IT to visit to reset their passwords. Unfortunately this coincided with a scheduled roll out of upgrades to the apps. This created a further burden for participants and highlighted the need for ongoing IT support.

### Change management considerations

There are also a variety of issues that need to be considered in implementing telehealth changes from a service perspective. Telehealth represents a new form of practice and staff require support in developing skills and changing work practices. Reluctance to change practice to a telehealth option for monitoring of patients may be due, in part, to fear of missing something that would otherwise be picked up from a physical home assessment even though home visits continued to occur during the trial. Clinicians need to see that the change has patient and carer benefits not just system or service benefits [30]. For some services early adoption of technology may be rewarding while for other services new technologies will add significant stress. Therefore active change management is an inevitable part of telehealth planning and implementation.

## Limitations of the study

There are a number of limitations that need to be acknowledged. The number of participants was relatively small and as it was a feasibility study there was no control group against which to compare outcomes. Recruitment related to a single area of service provision within a single metropolitan palliative care service. Participants were self-selecting. While the study model tried to minimise additional study support to mimic “normal” service activity, there was additional technical and study support available to the service. However, the recruitment and participation rates suggest that telehealth is of sufficient maturity to support properly constructed and powered trials.

## Conclusions

This study has shown that palliative care patients and their carers living in the community were able to manage the technology associated with a telehealth trial involving videoconferencing and remote monitoring of symptoms. Participants included old and very old patients. While some level of technology problems are probably inevitable, it is possible to design and develop integrated systems that can be used in the community. The telehealth model offers new ways of supporting care at the end of life in the community and further applications of these approaches should be investigated.

## Abbreviations

AKPS: Australia-modified Karnofsky Performance Status
AQoL: Assessment of Quality of Life
CAQ-CNS: Caregiver Assessment Questionnaire-Caregiver Network Service
CDS: Clinical decision support
CNF: Caregiver Network Facilitator
CPC: Nurse Clinical Practice Consultant
CPOE: Computerized provider order entry
GP: General practitioner
PCTRT: A Palliative Care Telehealth Research Team
SAPS: Southern Adelaide Palliative Care Service
SAS: Symptom Assessment Scale
